# Harrison C. Spencer Jr. (September 22, 1944, to August 10, 2016)

**DOI:** 10.4269/ajtmh.95-4obit

**Published:** 2016-10-05

**Authors:** Tony Mazzaschi

**Affiliations:** Association of Schools and Programs of Public Health (ASPPH), 1900 M Street NW, Suite 710, Washington, District of Columbia 20036 Email: tmazzaschi@aspph.org

**Figure 1:**
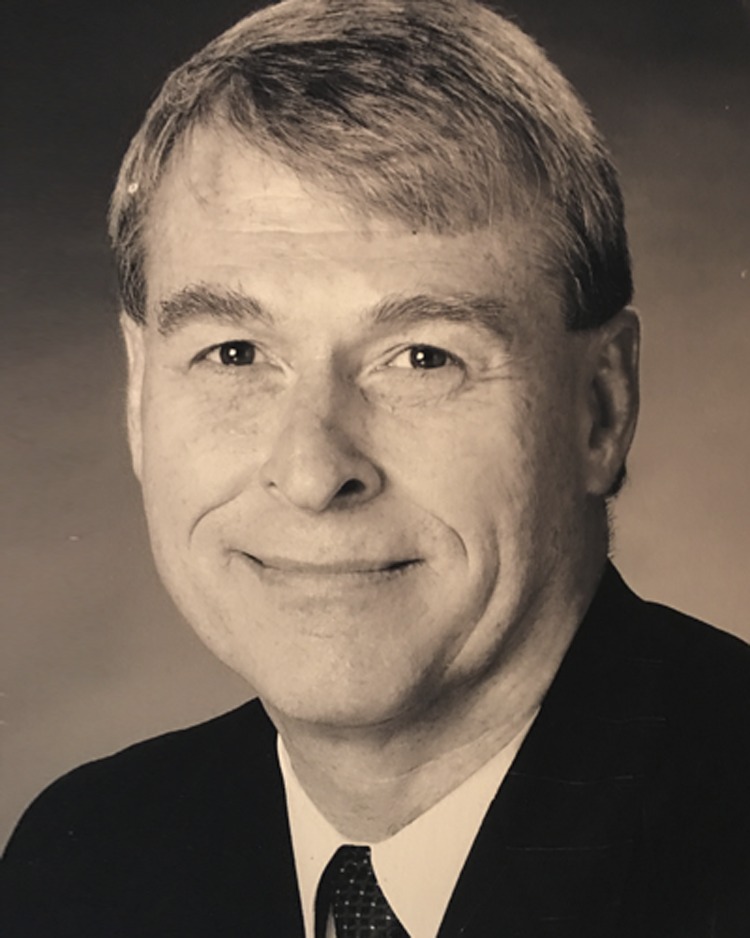

Harrison C. Spencer, Jr., MD, MPH, DTM&H, CPH, died on Wednesday, August 10, 2016, in Washington, DC. He is survived by his wife of 39 years, Christine M. Spencer, and two sons, Harrison (Trey) Spencer III, and Peter M. Spencer, daughter-in-law Katherine Spencer, and granddaughters Gigi and Maggie. He is also survived by two brothers and their wives, Fred and Jackie Spencer and Timothy and Ann Spencer. He was predeceased by his parents, Harrison C. Spencer, Sr., MD, and Dorothy (Stokes) Spencer and by a sister, Harriet Spencer Hoxton.

At the time of his death, Dr. Spencer was president and chief executive officer of the Association of Schools and Programs of Public Health (ASPPH). He joined the Association in 2000 when it was known as the Association of Schools of Public Health (ASPH). He led the ASPH's transformation to ASPPH in 2013.

Dr. Spencer was born at the Johns Hopkins Hospital in Baltimore on September 22, 1944. He lived in Baltimore until moving at the age of 6 to Abingdon, VA. After receiving his BS degree from Haverford College in 1965, Dr. Spencer received his MD degree from the Johns Hopkins University School of Medicine in 1969. Following an internship in medicine at Vanderbilt University Hospital in Nashville, he completed residencies in medicine and preventive medicine as a member of the U.S. Public Health Service in San Francisco and at the University of California, Berkeley, CA. While at Berkeley, he received an MPH degree from the School of Public Health. In 1972, he received a Diploma in Tropical Medicine and Hygiene from the London School of Hygiene and Tropical Medicine. After serving at the Centers for Disease Control and Prevention (CDC) in Atlanta, he returned to San Francisco to complete a residency in internal medicine at University of California, San Francisco. He subsequently became board certified in Internal Medicine and Preventive Medicine.

Dr. Spencer was affiliated with the U.S. Public Health Service and the CDC from 1970 to 1991. His service included appointments as Epidemic Intelligence Service Officer, CDC Atlanta, GA (1972–1974); Medical Officer, Central America Research Station, CDC, San Salvador, El Salvador (1975–1977); Medical Officer, Bureau of Tropical Diseases, CDC, Atlanta (1977–1979); Senior Physician and Malaria Coordinator, Clinical Research Center, Kenya Medical Research Institute, and Senior Lecturer, Department of Community Medicine, University of Nairobi Medical School (1979–1984); Senior Medical Officer, World Health Organization, Geneva, Switzerland (1984–1987); and Chief, Parasitic Diseases Branch, Division of Parasitic Diseases, CDC, Atlanta (1987–1991).

From 1991 to 1995, Dr. Spencer served as dean of the Tulane University School of Public Health and Tropical Medicine in New Orleans. He was a member of the American Society of Tropical Medicine and Hygiene, and served as a councilor from 1993 to 1995. He subsequently served as dean of the London School of Hygiene and Tropical Medicine, University of London from 1996 to 2000.

At the time of his death, Dr. Spencer was an adjunct professor in the Department of International Health at the Johns Hopkins Bloomberg School of Public Health and in the Department of Epidemiology at the Milken Institute School of Public Health at George Washington University. Earlier in his career, he held senior academic appointments at the London School of Hygiene and Tropical Medicine, Tulane University School of Public Health and Tropical Medicine, Tulane University Medical School, and the Morehouse School of Medicine.

Dr. Spencer was the author of dozens of scientific papers and book chapters published in leading peer-reviewed scientific journals and textbooks.

A member of the National Academy of Medicine (formerly the Institute of Medicine), Dr. Spencer was also a founding fellow of the UK Academy of Medical Sciences, an honorary fellow of the UK Faculty of Public Health Medicine, a fellow of the American College of Physicians, a fellow of the American College of Preventive Medicine, and a fellow of the Royal Society of Tropical Medicine and Hygiene. He received the U.S. Public Health Service Commendation Medal in 1984 and 1991 and the Outstanding Service Medal in 1989.

Throughout his long and distinguished career, Dr. Spencer served on dozens of committees and boards. At the time of his death, he was chair of the Interprofessional Education Collaborative and a member of the National Academy of Medicine's Leadership Consortium for a Value and Science-Driven Health System. He earlier had served as the chairman of the World Health Organization's Task Force on Malaria.

In addition to his immediate family, Dr. Spencer leaves countless public health professionals, academic public health leaders, and association colleagues inspired by his intellect, passion, leadership, and compelling ethical values.

